# Predation Danger Can Explain Changes in Timing of Migration: The Case of the Barnacle Goose

**DOI:** 10.1371/journal.pone.0011369

**Published:** 2010-06-30

**Authors:** Rudy M. Jonker, Götz Eichhorn, Frank van Langevelde, Silke Bauer

**Affiliations:** 1 Resource Ecology Group, Wageningen University, Wageningen, The Netherlands; 2 IPHC-DEPE, Université de Strasbourg, Strasbourg, France; 3 CNRS, Strasbourg, France; 4 Department of Animal Ecology, Netherlands Institute of Ecology (NIOO-KNAW), Maarssen, The Netherlands; 5 Swiss Ornithological Institute, Sempach, Switzerland; University of Bristol, United Kingdom

## Abstract

Understanding stopover decisions of long-distance migratory birds is crucial for conservation and management of these species along their migratory flyway. Recently, an increasing number of Barnacle geese breeding in the Russian Arctic have delayed their departure from their wintering site in the Netherlands by approximately one month and have reduced their staging duration at stopover sites in the Baltic accordingly. Consequently, this extended stay increases agricultural damage in the Netherlands. Using a dynamic state variable approach we explored three hypotheses about the underlying causes of these changes in migratory behavior, possibly related to changes in (i) onset of spring, (ii) potential intake rates and (iii) predation danger at wintering and stopover sites. Our simulations showed that the observed advance in onset of spring contradicts the observed delay of departure, whereas both increased predation danger and decreased intake rates in the Baltic can explain the delay. Decreased intake rates are expected as a result of increased competition for food in the growing Barnacle goose population. However, the effect of predation danger in the model was particularly strong, and we hypothesize that Barnacle geese avoid Baltic stopover sites as a response to the rapidly increasing number of avian predators in the area. Therefore, danger should be considered as an important factor influencing Barnacle goose migratory behavior, and receive more attention in empirical studies.

## Introduction

In migratory species, flexibility allows dealing with a continuously changing environment. Illustratively, Sutherland [1] presented an overview of bird species that showed flexibility in their migratory behavior to changing environmental conditions. He described changes in the use of wintering, breeding and staging areas, occurring in a wide range of families. Recently, Jonzén et al. (2006) suggested a climate-driven evolutionary change in the timing of spring migration for a number of long-distance passerine migrants [2] but see [3]. Changes in migration can also be caused by factors other than climate. Gill et al. [4] for example, showed that an increasing population of Black-tailed godwits *Limosa limosa islandica*, wintering in the UK, established new wintering sites on less suitable sites than the original wintering sites. They suggested that the carrying capacity of the original sites was reached, forcing the Black-tailed godwits to winter elsewhere. Additionally, Klaassen et al. [5] adopted a dynamic state variable model and showed that Pink-footed geese *Anser brachyrhynchus* respond to scaring practices by farmers in Norway by changing their use of stopover sites. Alerstam & Lindström [6] discussed minimization of time, energy and predation during migration as the main drivers of evolution in migratory behavior. The aforementioned examples of migratory change might represent responses to changes in one or more of these factors. Identifying possible causes of these changes, is essential for understanding flexibility in migratory behavior.

Since the early 1990s, an increasing number of Barnacle geese *Branta leucopsis* breeding in the Russian Arctic have delayed their departure from their wintering site in the Netherlands by approximately one month. The geese reduced their staging duration in the next stopover area in the Baltic (traditionally used by the entire population) according to the delay from the Netherlands, such that some migrants virtually skip the Baltic stopover site altogether [7], [8]. Because of these changes, the question arose what has caused the delayed departure from the wintering site and decreased use of the Baltic stopover site. Compared to changes in (migration) phenology in other bird species [2], [9], [10], [11], the rate of change of approximately 3 days/year as observed in the Barnacle goose is unprecedently large. One important consequence of the delayed migration of Barnacle geese is an increased agricultural damage in the Netherlands of approximately €350,000 annually, and this figure is growing rapidly [12]. Successful management actions require the identification of factors and processes affecting departure and staging decisions. Therefore, we have formulated three possible explanations for the delay: Barnacle geese have delayed their departure as a consequence of changes in (i) onset of spring, (ii) potential food intake rates, and (iii) predation danger [13].

### (i) Advanced onset of spring

Recently, several studies have found that migratory birds responded to climate-driven changes in plant phenology with advanced laying dates [14], advanced spring arrival dates [2], [10], [15] or increased rate of spring migration [9]. Climate change could result in higher spring temperatures in some regions, leading to earlier growth of the vegetation. Barnacle geese are thought to schedule their migration according to the “green wave” of fresh plant growth along the flyway [16]. However, this relationship might not be that straightforward, because geese may prioritize other factors, such as safety or food quality. Therefore, the potential effect of onset of spring is investigated in this study.

### (ii) Decreased intake rate

The potential intake rate at a stopover site, i.e. the intake per day a goose can gain if foraging at maximum intensity, limits the rate at which geese can replenish their energy reserves [17]. Earlier studies have shown that decreased availability and reduced quality of food can make a stopover site less attractive [18]. Van der Graaf [19] reported lower intake rates in the Baltic as compared to the Netherlands. Moreover, as the total population of Barnacle goose passing through the Baltic has increased drastically over the past thirty years [20], the competition for food at the Baltic stopover site may also have intensified [21]. Additionally, desertion of farmland, and thus reduced facilitation by cattle grazing, in these regions may also have decreased intake rates [22]. For these reasons, decreased potential intake rates at the Baltic stopover site may cause Barnacle geese to reduce staging time or even completely skip this site. Then, the geese could fly directly to one of the next stopover sites in Russia; however, since food there becomes available only later in spring, they have to delay their departure from the Netherlands until spring starts in the arctic stopover sites in Russia.

### (iii) Increased predation danger

Increased predation danger can reduce the attractiveness of a site because of its lethal and non-lethal effects [23], [24]. Although safety has long been acknowledged as potentially important for successful migration [6], it has received little attention so far and the few studies on the impact of predation danger on migration have not led to unambiguous conclusions [25], [26]. While a number of studies indeed demonstrated the effects of predators on body mass, stopover duration and site usage [27], [28], some of the results are difficult to interpret [29], and others even deny at least some of the suggested effects of predation danger [30].

In this study, we used a dynamic state variable model to analyze whether these three hypotheses can explain the observed changes in migratory behavior of Barnacle geese.

## Methods

We used a dynamic state variable model to predict the migration strategy of the Barnacle goose that maximizes expected lifetime reproductive success under different environmental circumstances. This type of model is most suitable as it includes future goals (maximising long term reproductive success) when defining decisions that lead to achieving these goals [31], [32]. We used an already existing model (see for more details [5], [33], [34]) which we parameterized for the Barnacle goose. We shortly explain the model here to give insight in the logic of the used parameters and to facilitate understanding our predictions.

### The dynamic state variable model

**Figure 1:**
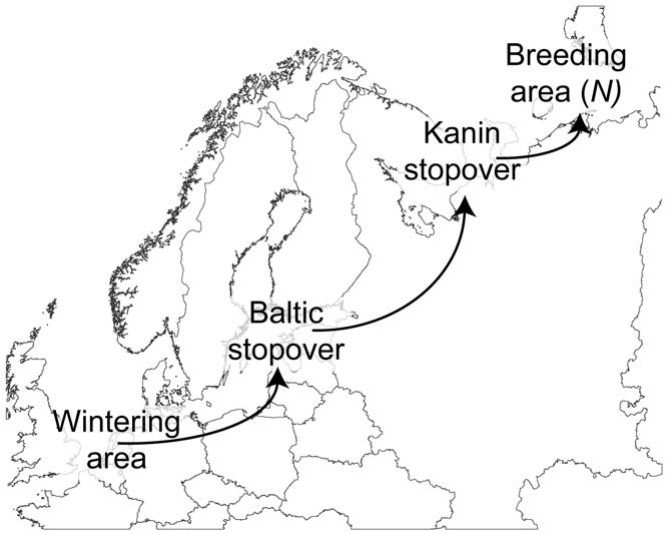
Migration route of Russian Barnacle goose. A schematic overview of the flyway of the Russian population of the Barnacle Goose. In spring (April–May), Barnacle geese depart from The Netherlands to stopover in the Baltic. After a stop of a few days to a few weeks they depart to pre-breeding areas in Northern Russia. The geese arrive at their arctic breeding grounds early June and start breeding immediately.

The state of the goose in the model was characterized by its energy stores *x* and its location *i*. At each time step of one day, *t* = 0,1…*T*, the state of body reserves was calculated, and according to state, location and time decisions for optimal migration was made. For computational reasons, *x* took only integer values between 0 and *x_max_* = 100. One unit of *x* was equivalent to 232 kJ, representing 1% of the caloric value of the maximum body reserves (see table 1 for an overview of parameters). If the body reserves fell to zero, the goose died of starvation. We considered 4 different locations: a wintering site in the Netherlands, stopover sites in the Baltic sea region and at the Kanin peninsula in Russia, and a breeding site *N* at the Barents Sea coast in Russia [35] (figure 1). Breeding was only possible at the breeding site. At *t* = 0 (March 1) the goose started at the wintering site and simulations ended when it reached the breeding site or when *t* reached *T*, a predefined endpoint which was set to *t* = 121 (June 29), approximately 3 weeks after the optimal time window for breeding. The expected reproductive success of the goose, with body reserves *x* at time *t* at location *i*, was denoted by *F*(*x*,*t*,*i*).

**Table 1:** Parameterization of the model.

Model parameters Barnacle geese			
Parameter		unit	Reference
Lean body mass	1500	g	Eichhorn 2008
Maximum body mass	2300	g	Eichhorn 2008
Potential mass reserves	800	g	
Energy density	29	kJ/g	Madsen and Klaassen, 2006
Total energy reserves *x_max_*	23.2	MJ	
Energy density per x	232	kJ	
Flight speed *v*	18	m/s	Green, 2001
Average flight costs *f*	6.23	kJ/km	Butler et al., 2000; Nolet et al., 1992; Ward et al., 2002
Daily energy expenditure *e*	4.7	kJ	Bruinzeel et al., 1997
Model parameters of the staging areas of the Russian flyway			
**Wintering site The Netherlands**			
Distance to wintering site	0	km	
Maximum metabolizable energy intake *g*	1397	kJ/day	Eichhorn 2008
**Stop-over site Baltic**			
Distance to wintering site	1270	km	
Maximum metabolizable energy intake *g*	1939	kJ/day	Eichhorn 2008
Peak date of food availability	May 14		Van der Graaf et al., 2006
**Stop-over site Kanin**			
Distance to wintering site	2910	km	
Maximum metabolizable energy intake *g*	2296	kJ/day	Eichhorn 2008
Peak date of food availability	May 20		
**Breeding site Kolokolkova Bay**			Van der Jeugd et al., 2003
Distance to wintering site	3270	km	
Time-window of arrival for optimal arrival K(t)	June 5–June 10		Eichhorn et al., 2006

#### *Terminal reward function*

The terminal reward was defined as the reward at *T*, and served as a starting point for the backward iteration. Upon arrival at the breeding site *N* the expected reproductive success *F*(*x*,*t*,*N*) depended on the body stores at arrival as well as the timing of arrival [36]. Additionally, a component was added for expected future reproductive success *B_T_* because Barnacle geese are long-lived animals with many years of breeding attempts. Thus:
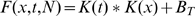
(1)where *K(t)* was the function of the timing of arrival, *K(x)* the function of the body stores on arrival, and *B_T_* was set to 2, representing the expected future reproductive success given that an individual actually survived at any site until *T*. Both *K(t)* and *K(x)* result in 0 reward if an individual had not arrived at breeding site *N* at *T*. Subsequently, the effect of timing of arrival was incorporated by a step function, meaning that breeding was only possible if arriving at the breeding grounds within the set time-limits:
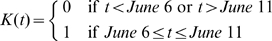
(2)
[20], [36]. The effect of body reserves on breeding success was described by a sigmoidal shape function based on data from the Pink-footed goose [36], indicating that the chance of successful breeding success increased if body stores upon arrival at the breeding site exceeded a certain threshold *x_c_*. We assumed a similar relationship for Barnacle geese. Thus:
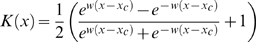
(3)where the shape parameter *w* was set to 0.028 and *x_c_*, the threshold for successful breeding, was set to 15080 kJ (*x_c_* = 65)

#### *Backward iteration*

At each time step a goose decided whether to stay at its present location and forage, or to depart to another location. When staying at location *i*, the potential intake rate (defined as metabolizable energy intake according to [37]) of the goose was site- and time-dependent and had predefined stochasticity [*g*(*i*,*t*), kJ day^−1^]. However, the actual intake rate depended on the foraging intensity *u*, ranging from 0 (no foraging) to 1 (continuous foraging). The actual intake rate minus the energy expenditure *e* [kJ day^−1^] resulted in the energy available for the storage of reserves. However, foraging with a particular intensity and storing reserves had a cost in terms of predation risk, defined by *β*(*x*,*u*):
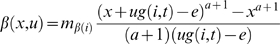
(4)where *a*, the mass-dependent escape performance exponent, was set to 2 and the site-specific constant attack rate [33] *m_β(i)_* is set to 10^−8^. The parameter *m_β(i)_* is the predation danger according to the definition by [13]. Thus, the goose foraged with the intensity that maximized its expected reproductive success *F*:
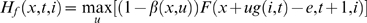
(5)


Alternatively, when departing to another site *j*, the goose chose the site *j* that maximized *F*:
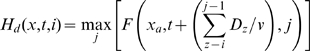
(6)This choice depended on the distance between the sites [*D_z_* (km)], the speed of flight [*v* (km day^−1^)], and the reserves upon arrival (*x_a_*) at site *j*. The latter was defined by
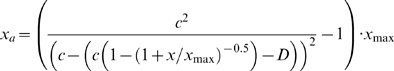
(7)where *D* was the distance covered. The constant *c* in this equation was defined by
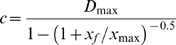
(8)where *x_f_* was the level of body reserves available for flight, which equaled *x_max_* for Barnacle goose, and *D_max_* was the maximum flight distance defined by
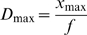
(9)where *f* was the average flight cost [kJ km^−1^] [38], [39], [40]. To find the fitness-maximizing decision, we calculated the fitness consequences of the behavioral alternatives, i.e., to forage or depart, for all combinations of state, location and time and chose the one with the highest fitness. The thus obtained optimal decision matrix showed the best decision for each time step and for all possible levels of body reserves and sites, namely:
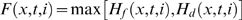
(10)


#### *Forward simulation*

Based on the decision matrix, optimal migration was simulated for each goose. The simulations started at *t* = 0, each goose started with a random amount of body reserves between 4640 kJ≤x≤11600 kJ, and ended when the bird reached the breeding site, died, or passed the time limit *T* at any other site. In the simulations, we assumed geese had full knowledge of the environment, i.e. the geese experienced the same conditions in the forward simulation for which the optimal decisions were calculated in the backward calculation. The actual experienced potential intake rate *g*(*i*,*t*) for each individual was drawn from a distribution with a predefined stochasticity.

### Scenarios

We analyzed the three different hypotheses by step-wisely changing the relevant model-parameters, i.e., onset of spring, intake rates and predation danger. For all scenarios, both backward iteration and forward simulations were run. First, we changed onset of spring in the Baltic staging site from 24 April to 3 June in steps of 5 days. Onset of spring was defined as the point in time when food availability *g*(*i*,*t*) first reached its highest value. Second, we changed food availability in the wintering and Baltic stop-over site from 1392 kJ d^−1^ to 2784 kJ d^−1^ in steps of 232 kJ d^−1^, and in all possible combinations for both sites.

Third, we increased predation danger (*m_β(i)_*) in the Baltic site from 10^−10^ to 10^−6^ with 16 logarithmically equal steps (10^−10^, 10^−9.75^, 10^−9.5^, …, 10^−6.5^, 10^−6.25^, 10^−6^). We choose this range of values based on the value of 10^−8^ used by Klaassen et al. [5] and the value of 2·10^−6^ used by Weber et al. [33].

**Figure 2:**
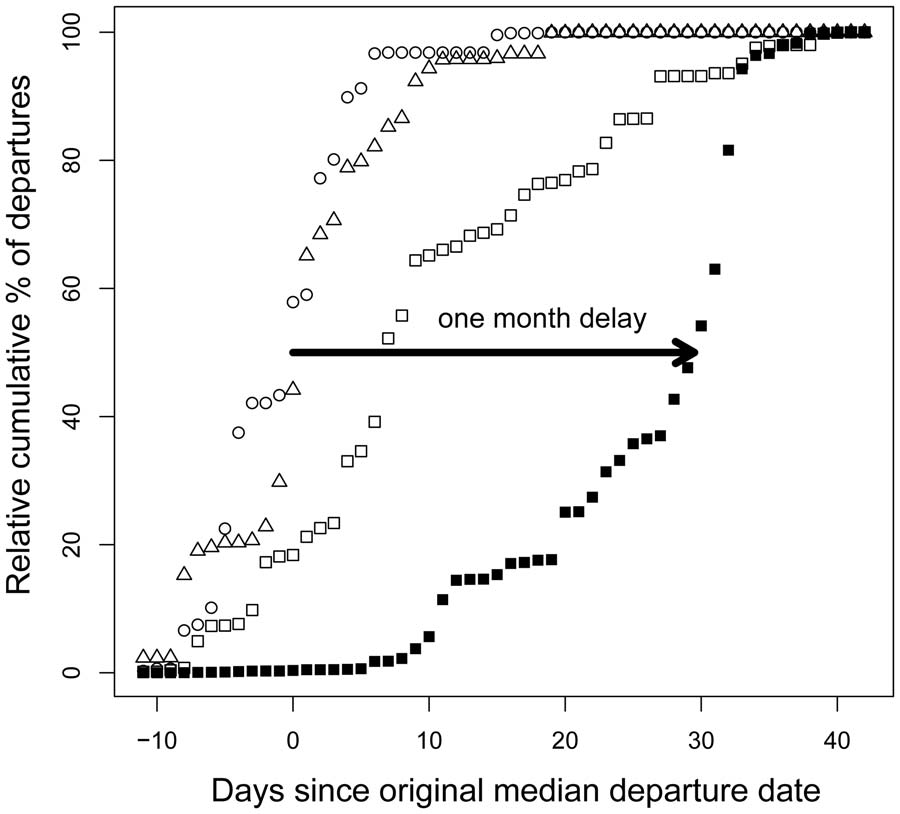
Observed delay in onset of spring migration. The departure dates from the wintering grounds in the Netherlands, shown as the relative cumulative percentage of departure as a function of days since the median departure date in the 1970's. Data points represent per day the mean relative cumulative passage count at Ottenby bird observatory over a certain period (circles: 1970–1979, triangles: 1980–1989, open squares: 1990–1999, solid squares: 2000–2004). The median departure date in the 1970's was April 12.

We compared the model predictions of the three scenarios with passage data from the Ottenby bird observatory (56°11′45″N, 16°23′56″E) from 1970 until 2004 (adapted from [41], see figure 2). Ottenby is situated on a main migratory corridor for Barnacle geese traveling from the Netherlands to Baltic stopover sites [42]. Because the total population of Barnacle geese also greatly increased during that period, we used the relative cumulative percentage of passed dates. The most plausible predictions were those that showed a delay in departure equivalent to the observed delay of one month. All results were analyzed with R.2.8.1 [43].

## Results

**Figure 3:**
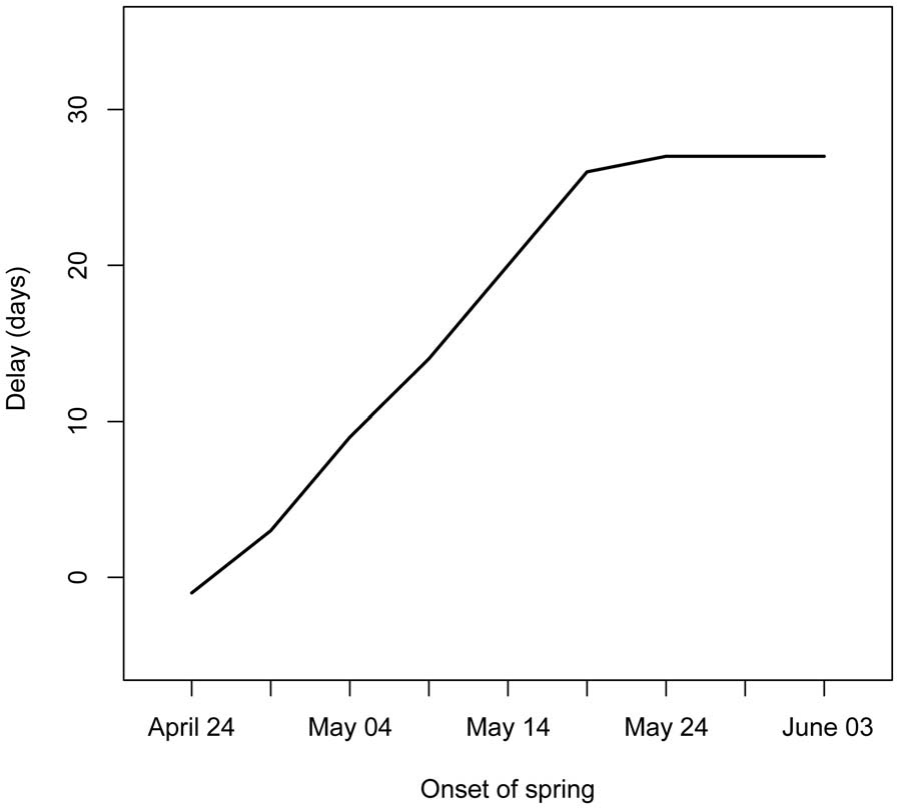
Predicted delay in onset of spring scenario. The delay in departure (in days since April 12, which was the median departure date in the 1970's) from the wintering site in the Netherlands as a function of onset of spring. In the model, the geese responded to a change in the peak date of intake rate such that they advance departures with an earlier spring and vice versa, they would depart later from the wintering site if spring in the Baltic would be delayed.

Advancing the onset of spring in the Baltic by a given unit of time led to an equally advanced departure date from the wintering site for most of the range tested in our simulations (figure 3). Additionally, the simulations showed that the geese always depart from the Dutch wintering site just before the onset of spring in the Baltic.

**Figure 4:**
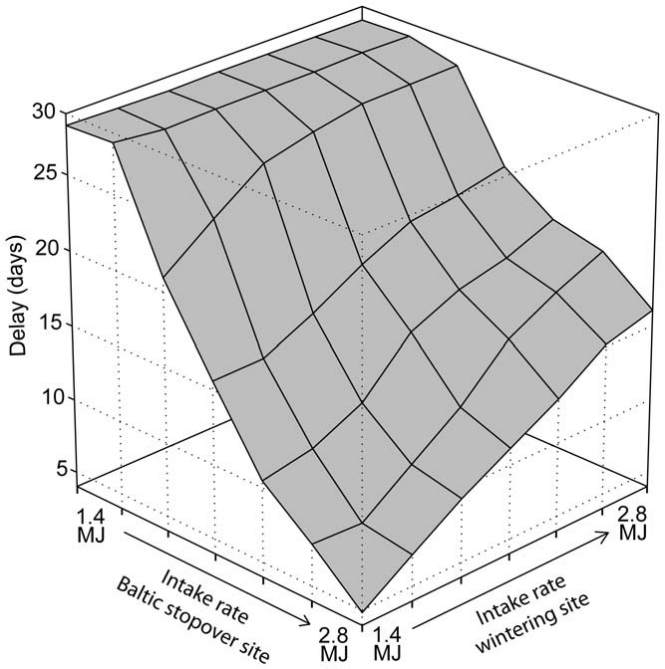
Predicted delay in intake rate scenario. The predicted delay in departure date (in days since April 12, which was the median departure date in the 1970's) from the wintering site in the Netherlands to a changed intake rate, ranging from 1.4 MJ to 2.8 MJ, at the wintering site and the Baltic stopover site.

Decreasing intake rates in the Baltic stopover sites by 1392kJ/day led to a delay in departure date from the wintering site of 29 days (mid April–mid May) (figure 4). If, alternatively, the intake rates in the wintering site increased, the geese delayed their departure date by only 16 days (figure 4).

**Figure 5:**
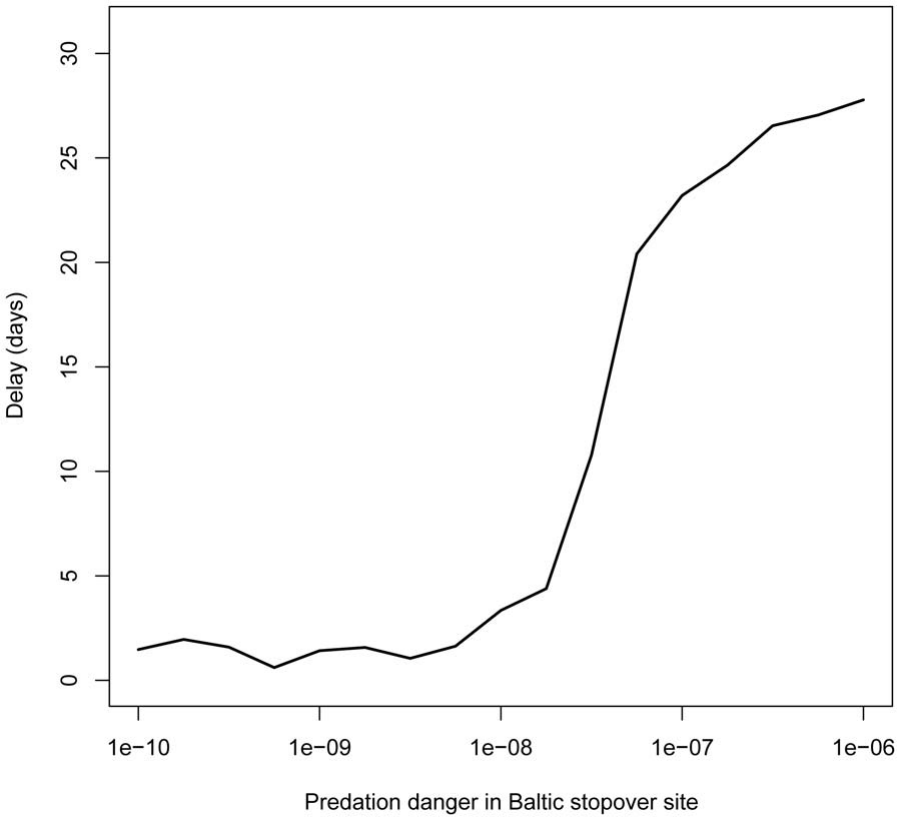
Predicted delay in danger scenario. The delay in departure (in days since April 12, which was the median departure date in the 1970's) from the wintering site in the Netherlands as a function of predation danger at the Baltic stopover site. Above a predation danger of 3·10^−8^, the geese adjusted their migration by abruptly delaying their departure date from the wintering site by up to 28 days.

**Figure 6:**
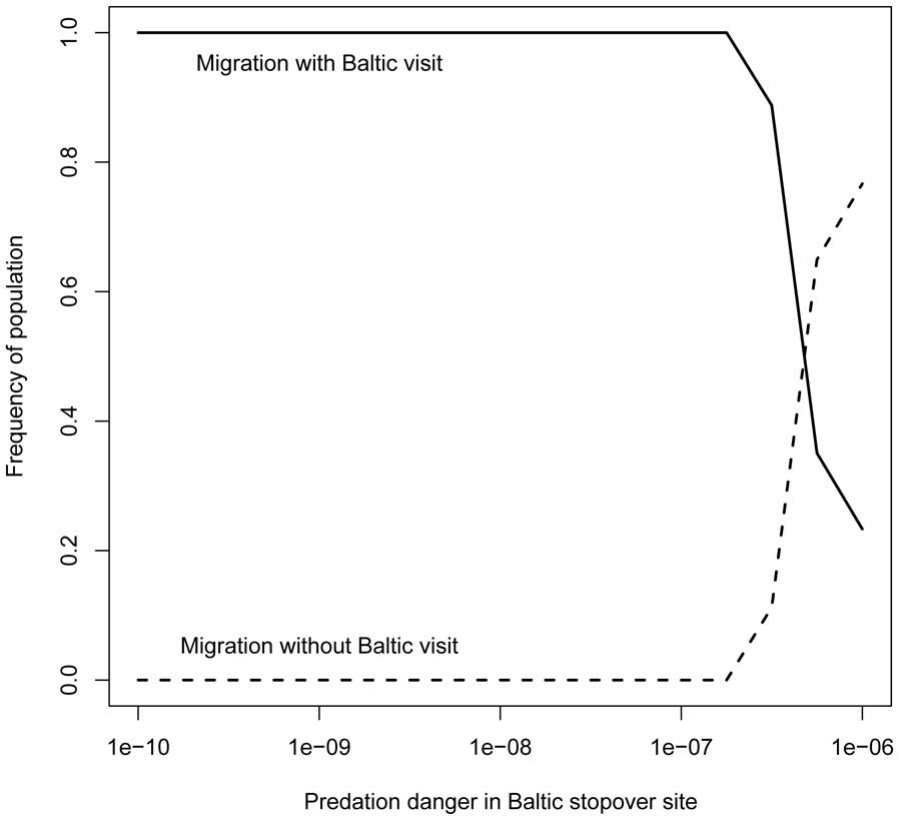
Predicted use of Baltic stopover site in danger scenario. The predicted response to increased predation danger, described as the proportion of the geese that make use of the Baltic as a stopover site. With low predation danger all geese are predicted to use the Baltic stopover site (solid line), i.e. no skipping of the Baltic (broken line). However, with increasing predation danger the majority (+/−75%) of the geese skip the site while some geese with (very) low body reserves continue to use the Baltic stopover site for a few days to build up extra reserves.

Increasing predation danger in the Baltic above the predation danger of the other sites led to a rapid delay of 28 days (mid April–mid May) in departure date from the wintering site (figure 5). When predation danger was further increased, a growing proportion of geese stopped using the Baltic stopover site (figure 6). However, a small proportion geese still visited the Baltic, and stayed for a few days only. They had low energy reserves, and apparently, could not skip this site as they were in dire need of replenishing their body stores.

## Discussion

Our simulations showed that the delayed departure of Barnacle geese from their wintering grounds by up to one month can be explained by either decreased potential intake rates or increased predation danger in the Baltic stopover site. In contrast, an advanced onset of spring fails to explain such a delay. The predicted response to an advanced spring growth is opposite to a delayed departure actually observed in the field. According to our simulations, an advancement of spring of 8 days (as predicted by [19] based on growing degree days) should advance departure by 8 days too. Interestingly, also the Barnacle geese breeding on Spitsbergen have not advanced their departure from Scottish wintering grounds despite an advanced onset of spring at their Norwegian stopover site, in contrast to Pink-footed geese, which largely share the same flyway and have advanced their spring migration [44]. Tombre et al. [44] suggest that Barnacle geese breeding at Spitsbergen cannot predict spring in Norway from their wintering site in the United Kingdom because of the large overseas crossing. The Russian breeding Barnacle geese, however, do not have such a large overseas crossing, and prioritize other factors than responding to advanced onset of spring in the Baltic. Thus, although the timing of high quality food during migration is important for Barnacle geese [16], this result suggests that Barnacle geese may prioritize other factors above the onset of plant growth in spring, and that the observed delay in migration cannot be caused by climatic changes. Theory also predicts that birds should not advance their timing of migration as much as spring advances, because the timing of migration has not only evolved to match the peak of food availability but also in response to many other factors, such as competition for territories and predation risk [45].

Our assumptions on decreased potential intake rates are supported by empirical studies [16], [41]. Both studies suggested a recent decrease in intake rates in a Baltic stopover site. Additionally, Barnacle geese have been observed to colonize new staging sites at several locations in the Baltic. Populations staging at traditional sites remained approximately constant [46], indicating that the traditional sites reached capacity, especially because the total population of geese increased much more than the population staging in the Baltic [7]. Besides, the ongoing urbanization in the Baltic region has led to a general decline in agricultural practice, e.g., cattle farming. Consequently, intake rates may also have decreased as facilitation by large grazers decreased. Altogether, decreased intake rates can be a plausible explanation for the observed delay.

In addition to the importance of food *en route*, our simulations showed a particularly strong effect of predation danger on the departure date from the wintering site. When predation danger in the Baltic was only slightly higher compared to the other sites, the geese immediately started delaying departure from the wintering site, reducing staging time at the dangerous site and ultimately, skipping the site with higher predation risk. This is in line with theoretical predictions that a migratory bird should minimize the time spent in a dangerous area [47] and that the loss of future reproductive success by predation is traded off against the benefit of increasing reserves by foraging [48]. Predators can have a strong influence on migratory strategies, e.g. by causing migrants to avoid the predator abundance peak [28]. If the whole Baltic area has become more dangerous due to the recovery of predator populations, we expect the geese to minimize the time spent in that area. The strong increase in predator numbers such as White-tailed eagles in the Baltic; a fourfold increase in Estonia (from 40 to 150–170 [49]), Latvia and Finland and expansion into Gotland, Sweden [50], indicates that the Baltic has indeed become a more dangerous place for Barnacle geese compared to the Netherlands. For example, on the island of Saaremaa (2,672km^2^), Estonia, which is a major stopover site in the Baltic, there are 28 known White-tailed eagle territories (pers. comm. V. Völke). Contrastingly, there is currently only a single breeding pair in the Netherlands (41,528km^2^). For this breeding pair it has been confirmed that it preys on Greylag geese *Anser anser* [51].

Additionally, predation danger caused birds to not take full advantage of available resources, as they take the danger into account in their decision of where to forage [52]. These non-lethal effects of predation can potentially be larger than the lethal effects [24]. Hence, increased predation danger can reinforce the already existing effects of decreased intake rates. The influence of density-dependent effects on this trade-off are not immediately clear. Potentially, danger can cause many geese to shift to safer areas, thereby decreasing the competition for food in the dangerous areas. However, it is known that Barnacle geese facilitate each other while grazing [53]. Consequently, a dangerous and less grazed area does not necessarily lead to better feeding conditions. Our model did not take these density-dependent effects into account.

In conclusion, predation danger, in addition to food availability, can be a key factor in explaining the observed changes in migratory behavior of Barnacle geese. This study only approached the problem from a theoretical point of view, but identified critical factors to be studied empirically in the field. These new insights also suggest that challenging geese with natural predators in the Netherlands, e.g. by creating suitable nesting places for White-tailed Eagles, may improve management of the agricultural conflict. Future empirical research needs to test our predictions by measuring the direct and indirect effects of predator activities on goose behavior. Although this study focused on the case of the Barnacle goose, its conclusions are not limited to goose migration. It is often assumed that timing of migration is synchronized with the phenology of resources [11], resulting in potential mismatches and associated population declines as a result of climate change [54]. These two studies state respectively that looking at predation in addition to resources as explanatory factor is very difficult or do not even mention predation at all as potential explanatory factor. We want to emphasize that in addition to currently well studied factors such as food availability and climatic change, predation danger should be considered in the suite of potential explanatory variables for changes in the migratory behavior of birds.
